# Review of insecticide resistance and behavioral avoidance of vectors of human diseases in Thailand

**DOI:** 10.1186/1756-3305-6-280

**Published:** 2013-09-25

**Authors:** Theeraphap Chareonviriyaphap, Michael J Bangs, Wannapa Suwonkerd, Monthathip Kongmee, Vincent Corbel, Ratchadawan Ngoen-Klan

**Affiliations:** 1 Department of Entomology, Faculty of Agriculture, Kasetsart University, Bangkok 10900, Thailand; 2 Public Health & Malaria Control Department, International SOS, Kuala Kencana, Papua 99920, Indonesia; 3 Department of Disease Control, Ministry of Public Health, Chiang Mai 52000, Thailand; 4 Department of Entomology, Faculty of Agriculture at Kamphaeng Saen, Kasetsart University, Kamphaeng Saen Campus, Nakhon Pathom 73140, Thailand; 5 Institut de Recherche pour le Développement (IRD), Maladies Infectieuses et Vecteurs, Ecologie, Génétique, Evolution et Contrôle (MIVEGEC, IRD 224-CNRS 5290 UM1-UM2), Montpellier, France

**Keywords:** *Anopheles*, *Culex*, *Aedes*, Control, Insecticide, Susceptibility, Behavior, Thailand

## Abstract

Physiological resistance and behavioral responses of mosquito vectors to insecticides are critical aspects of the chemical-based disease control equation. The complex interaction between lethal, sub-lethal and excitation/repellent ('excito-repellent’) properties of chemicals is typically overlooked in vector management and control programs. The development of “physiological” resistance, metabolic and/or target site modifications, to insecticides has been well documented in many insect groups and disease vectors around the world. In Thailand, resistance in many mosquito populations has developed to all three classes of insecticidal active ingredients currently used for vector control with a majority being synthetic-derived pyrethroids. Evidence of low-grade insecticide resistance requires immediate countermeasures to mitigate further intensification and spread of the genetic mechanisms responsible for resistance. This can take the form of rotation of a different class of chemical, addition of a synergist, mixtures of chemicals or concurrent mosaic application of different classes of chemicals. From the gathered evidence, the distribution and degree of physiological resistance has been restricted in specific areas of Thailand in spite of long-term use of chemicals to control insect pests and disease vectors throughout the country. Most surprisingly, there have been no reported cases of pyrethroid resistance in anopheline populations in the country from 2000 to 2011. The precise reasons for this are unclear but we assume that behavioral avoidance to insecticides may play a significant role in reducing the selection pressure and thus occurrence and spread of insecticide resistance. The review herein provides information regarding the status of physiological resistance and behavioral avoidance of the primary mosquito vectors of human diseases to insecticides in Thailand from 2000 to 2011.

## Introduction

A number of insect species can transmit pathogens to humans resulting in significant morbidity and mortality as well as placing a profound burden on human productivity and development. Transmission of these vector-borne diseases is related to the complex interplay of three primary components; pathogenicity/virulence of the infectious agent, vector competence (infectivity) and host (human) susceptibility. This transmission cycle is directly and indirectly driven by a diverse number of inter-related environmental factors. Successful control of human diseases requires an understanding of the interaction among these three components and the various other biological, environmental, and socio-economic factors that influence transmission. Such a task often requires or benefits from the full participation of both governmental and private sectors, sufficient numbers of trained personnel, adequate and sustained financial support, and a well-designed, evidence-based vector control program. Despite decades of organized vector control efforts, malaria, dengue, lymphatic filariasis and Japanese encephalitis, remain real threats in various areas of Thailand. One of the most effective means of prevention of these diseases involves vector control to reduce the risk of transmission. In some instances, this requires the use of various chemical compounds as larvicides applied to aquatic habitats and adulticides as outdoor space applications and indoor residual sprays (IRS), and the use of insecticide-treated bed nets [1-4] in order to reduce vector survival and density and thus human-vector contact.

At least four groups of synthetic compounds, organochlorine (DDT), organophosphates, carbamates and pyrethroids, have been extensively used in Thailand for the control of both agricultural pests and human/animal disease vectors. DDT was introduced for agricultural pest control in 1934 but was later banned from all agricultural use in 1983. For public health, the use of DDT was launched in 1949 in an indoor residual spray (IRS) pilot campaign to control malaria transmission after Thailand agreed to participate in the malaria eradication program outlined by the World Health Organization (WHO) [5-7]. However, the use of DDT began a gradual decline in use in the later decades and was completely removed for malaria control in the year 2000 due to its perceived adverse impact on the environment and declining public acceptance for indoor residual spraying [7-9]. Before 2000, the extensive use of DDT resulted in the development of physiological resistance in populations of *Anopheles aconitus*, *Anopheles culicifacies*, *Anopheles nivipes* and *Anopheles philippinensis*, all non-malaria vector species in Thailand [8].

For many decades, various synthetic insecticidal compounds have been used extensively in the private sector, agri-business and in the national public health vector control programs in Thailand. The vast majority of compounds, by number and volume, are pyrethroid-based formulated combinations (Table 1). Synthetic pyrethroids have become the most popular and prevalent active ingredients for public health use due to their relatively low mammalian toxicity but high invertebrate potency at low levels, resulting in rapid immobilization ('knockdown’) and killing [10]. Most of them have been used to control insect pests such as cockroaches, ants, bedbugs, and mosquitoes [11]. Compared to pyrethroids, relatively few organophosphate and carbamate-based insecticides remain available in the Thai public market. For example, the amount of pyrethroids used for dengue and malaria control in 2007 (excluding treated bed nets) was approximately 1,127 tons whereas that of OP and carbamates together was virtually nil [11].

**Table 1:** Twenty-three commercial products and active ingredients for household pest control in Thailand (2012)

**Product name (Trade name)**	**Compounds**	**Concentration**
Shieldtox (odorless I)	Bioallethrin	0.241%w/w
	Bioresmethrin	0.046%w/w
Shieldtox (odorless II)	Prallethrin	0.0729%w/w
	Phenothrin	0.1003%w/w
Shieldtox (Ultra I)	Bioallethrin	0.209%w/w
	Bioresmethrin	0.039%w/w
Shieldtox (Ultra II)	Tetramethrin	0.230%w/w
	Deltamethrin	0.015%w/w
Shieldtox (Ultra Green I)	Tetramethrin	0.230%w/w
	Deltamethrin	0.015%w/w
Shieldtox (Ultra Yellow 1)	Prallethrin	0.055% w/w
	Permethrin	0.100% w/w
	Tetramethrin	0.184%w/w
Raid Insect Killer 4	Propoxur	0.75% w/w
	Tetramethrin	0.30% w/w
	Cypermethrin	0.10%w/w
Raid X-tra	Tetramethrin	0.30%w/w
	Permethrin	0.10%w/w
	Transfluthrin	0.05%w/w
Raid X-tra Plus	Prallethrin	0.06% w/w
	Permethrin	0.20% w/w
Raid Insect Killer (water based)	Prallethrin	0.06% w/w
	Permethrin	0.24% w/w
Raid (water based)	Tetramethrin	0.35% w/w
	Allethrin	0.10% w/w
	Permethrin	0.10% w/w
ARS	Tetramethrin	0.07% w/w
	Dichlovos	0.50% w/w
ARS 3	Tetramethrin	0.02% w/w
	Permethrin	0.10%w/w
	Dichlovos	0.05%w/w
ARS (water based)	Allethrin	0.06%w/w
	Tetramethrin	0.06%w/w
	Permethrin	0.18%w/w
ARS Jet Pro	Imiprothrin	0.20%w/w
	Cypermethrin	0.10% w/w
BYGON (Blue)	Cyfluthrin	0.025% w/w
	Transfluthrin	0.040%w/w
BYGON (Yellow)	Transfluthrin	0.04%w/w
	Cyfluthrin	0.025%w/w
BYGON (Green)	Cyfluthrin	0.025%w/w
	Propoxur	0.500%w/w
	Dichlorvos	0.500%w/w
Kincho	Tetramethrin	0.20%w/w
	Permethrin	0.14%w/w
JUMBO	Bioallethrin	0.200%w/w
	Deltamethrin	0.012%w/w
	Permethrin	0.100%w/w
Sheldrite	Permethrin	0.25%w/w
	Bioallethrin	0.10%w/w
	Dichlovos	0.5%w/w
Atsawin	Tetramethrin	0.20%w/w
	Permethrin	0.10%w/w
GY-15	DEET	4%

The extensive use of pyrethroids for vector control has raised major concerns over the selection pressure induced by the insecticides on resistance gene mechanisms [4,12-15]. In addition to insecticides, topically applied repellents such as DEET (*N*, *N*-diethyl-*meta*-toluamide), one of the most effective insect repellent active ingredients [16,17], is available in most local markets and is used extensively to protect against biting mosquitoes and other insects (Table 1), despite potential negative health effects in humans associated with continuous or over application on the skin.

Insect resistance to insecticides has been observed for all classes of compounds, including microbial-based agents and insect growth regulators (hormone mimics) [18]. In general, response to insecticides can be categorized into two major types: physiological resistance and behavioral avoidance [19]. Physiological resistance is the ability of an insect population to survive exposure to a concentration of insecticide that would normally result in complete kill [1]. One or more mechanisms may be involved in physiological resistance, including alteration of target site nerve receptors (e.g., *kdr*, *Rdl* and *Ace*.*1R*) and detoxification via increased enzyme activity of non-specific esterases, glutathione *S*-transferases and P-450 mediated monooxygenases (mixed function oxidases) [20].

In contrast, behavioral avoidance (deterrence) is defined as the ability of an insect to move away (escape) from an insecticide-treated area, often without lethal consequence [4]. This type of response can be further divided into direct contact excitation (sometimes referred to as 'irritancy’) and non-contact spatial repellency [12]. The term 'contact irritancy’ involves an insect leaving an insecticide treated area only after making physical (tarsal) contact with the chemical, whereas 'spatial repellency’ is when insects move away from the insecticide-treated area without making direct contact [12,19]. Lastly, some chemicals, such as DEET, can elicit a fourth action by effectively masking/jamming the presence of a host through the inhibition of odor-activated receptors [21].

The review herein has compiled information on the use of public health insecticides in Thailand since 2000 and summarizes the primary insecticidal and behavioral responses of disease vector mosquitoes of importance elicited by these chemicals [8]. This should assist in guiding national authorities in the rational and target-specific use of insecticides for effective control of mosquito vectors.

## Review

### Insecticides used for the control of insect vectors and pests

Insecticides have been widely used to control both urban and peri-urban insect pests [1,2,22]. Although the use of DDT has been completely halted (banned) in many countries, recent allowances have been made for its renewed use in malaria control programs in a number of African nations because of its superior attributes compared to most alternative active ingredients [23]. The marked impact of DDT on mosquito populations in terms of both toxicity and modified behavioral responses that suppress disease transmission is well known despite lacking a clear understanding of the actual mechanisms and interactions at work in some instances. Most studies on insecticides have focused exclusively on the direct toxicological effects of the molecule on mosquitoes whereas comparatively fewer investigations have accurately measured the behavioral responses resulting from sub-lethal exposure to the active ingredient [12,19,24,25]. Observations on behavioral responses of vectors began with the early use of DDT to control *Anopheles* mosquitoes [1], avoidance outcomes which resulted in the recognition of two different types of non-toxic actions: excitation and repellency, often termed together as 'excito-repellency’ [1,4,26-33]. The importance (either benefit or drawbacks) of avoidance behavior without killing or reducing survival of the vector outright has produced plenty of debate and controversy for impact in controlling disease transmission [4,32].

In Thailand, many compounds have been used for the control of medically important insects in both the private and public sectors. In the private sector, several dispensing designs for household pesticides are available [13,14], including space sprays (aerosols), released as a vapor phase (mosquito coils, electric mats), direct applications (creams), and residual liquids. Often, these various formats contain more than one active ingredient and include synergists to enhance knockdown response and effectiveness. Over 80% of the active ingredients currently used in homes are pyrethroids that are used in low concentrations in the form of aerosols. By market volume, both organophosphates and carbamates (Table 1) are used to a far lesser extent. The most common use for home-based insecticides is for control of mosquitoes and other flying insects (house and filth flies) followed by termites, ants, cockroaches, and bedbugs. Among the pyrethroids, permethrin, deltamethrin and cypermethrin are the predominant active ingredients used (Table 1). In the business sector, pest control operators (PCOs) with proper training and special licensure, allows professionals to apply a wider (and more toxic) variety of chemicals to control a broader range of pests (e.g., structural pests such as termites and ants). Such PCO training programs, under the support and guidance of the Food and Drug Administrative Office, Ministry of Public Health, has been carried out by the Department of Entomology, Faculty of Agriculture, Kasetsart University at least twice each year since 2003. In the public (government) sector, a wider array of chemicals are used for vector control purposes including organophosphates, carbamates, pyrethroids and so-called 'bio-rational’ pesticides and biological agents, such as natural predators, bacterial toxins, insect growth regulators (hormone mimics) and botanical repellents, depending on the target species and circumstances [2,8,34]. For routine dengue vector control in Thailand, an organophosphate-based larvicide (temephos) has been commonly used for the control of *Aedes aegypti* larvae in container habitats since 1950 [8]. Although it remains mostly effective for *Aedes* larval control, evidence of temephos resistance in *Ae. aegypti* has recently been observed in some localities of Thailand [13]. The other organophosphates such as malathion, fenitrothion and pirimiphos-methyl were commonly used as either IRS or fogging agents before being replaced by pyrethroids [8]. In 1994, deltamethrin, a newer, more potent pyrethroid was introduced to Thailand for controlling indoor biting mosquitoes, including *Ae. aegypti*[8]. This compound remains the standard for the control of dengue vectors during dengue outbreaks although recent reports have identified deltamethrin resistance in several populations of *Ae. aegypti*[35]. Deltamethrin and permethrin have also been widely used in the malaria control program [36]. Deltamethrin is used for IRS once or twice a year, depending upon the intensity of malaria transmission in the area (based on endemic malaria zoning categories) determined by the Bureau of Vector Borne Disease [36]. Permethrin is still commonly used to impregnate fabric materials such as clothing, screens, blankets, and bed nets. These insecticide-treated materials can be more easily shipped to malaria endemic areas that may be difficult to access for spray teams because of relative isolation, poor roads and/or on-going civil insurgency and security disruptions. For example, in the four southern-most provinces of Thailand, cases of malaria have risen to nearly 4,000 a year in the area adjacent to the Thai-Malaysian border where rebels have been engaging Thai authorities since 2004. Similarly, the number of malaria cases reported along the Thai-Myanmar border (e.g., Tak and Mae Hongsorn provinces) has been aggravated due to the presence of refugee camps and intense migration from the adjacent country where malaria is still highly prevalent [37].

### History and organization of the vector-borne disease control program

**Figure 1:**
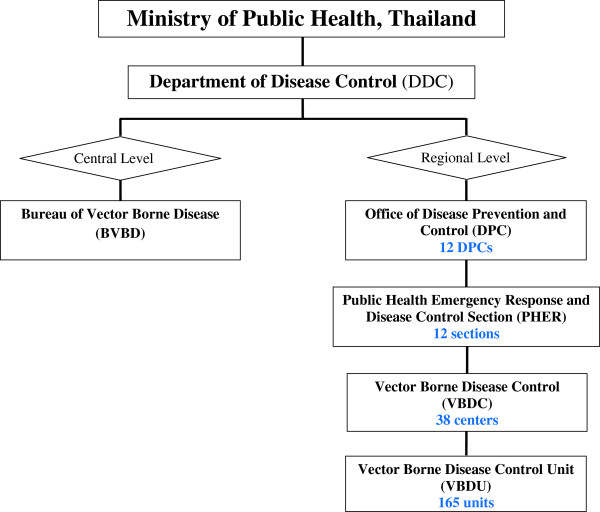
Diagram of the organization of the Vector-Borne Disease Control Program, Thailand.

Over the 60 year history of the malaria control in Thailand, a number of policy changes have occurred to adjust to new developments in technology and the shifting landscape of malaria epidemiology in the country. Since the implementation of the national malaria control program in the 1950s, malaria associated morbidity and mortality has been reduced dramatically in Thailand except along the border areas. However, in the last decade, a shortage of trained public health personnel and vector control specialists, together with flagging financial support has resulted in dramatic changes in the vector control policies in Thailand. In October 2002, the Department of Disease Control (DDC) reorganized the Vector-Borne Disease Control (VBDC) Program by merging the Malaria Control Unit (MCU) with other vector-borne disease control programs. The DDC was further consolidated to include other non-communicable diseases under its direction. The restructuring helped ease constraints on staffing, budget and equipment for all VBDC elements, and eliminated many of the redundancies and relatively high costs previously incurred by each of the former independent control programs. At the national level, the Bureau of Vector Borne Disease (BVBD) is under the direction of the DDC, in the Ministry of Public Health. The program comprises 12 regions under the direction of a Medical Officer, Director of the Office of Disease Prevention and Control (DPC). Under the DPC, the Vector-Borne Disease Section (VBDS) was set up to respond to the major vector-borne disease issues in each Regional DPC. In 2003, 39 Vector-Borne Disease Control (VBDC) Centers and 302 Vector-Borne Disease Control Units (VBDU) are set up at provincial and district levels, respectively, but recently these local VBD sectors were reduced to 38 VBDCs and 165 VBDUs, respectively. In addition, there are a number of district and sub-district municipalities that operate under the direction of the Ministry of Interior. In October 2011, the 12 DPC regional offices underwent further restructuring, and the VBDSs were merged with and renamed either Technical Support Sections or Emergency Response for Public Health Disasters, depending on the individual DPCs. This resulted in some confusion with the other Public Health Sectors with regard to knowing the correct individuals to contact for advice on insecticide usage and proper vector control application and monitoring techniques. This also included local district and sub-district administrative offices under the Ministry of Interior (Figure 1).

The Ministry of Interior is now responsible for developing their own policies for both vector and pest control activities, including planning and budget allocation. Little is done, however, in regard to pest control evaluation and monitoring. Moreover, each municipality has decision-making power for the local vector control operations, including budget allocations for purchasing insecticides. As a result, each local office can directly approach an insecticide company without a clear policy and evidence-based rationale for insecticide choice from the Department of Disease Control, MOPH. The majority of products purchased are typically pyrethroids that include deltamethrin, permethrin, cypermethrin, and alpha-cypermethrin (Wannapa, personal communications).

### Mosquito-transmitted diseases in Thailand

**Figure 2:**
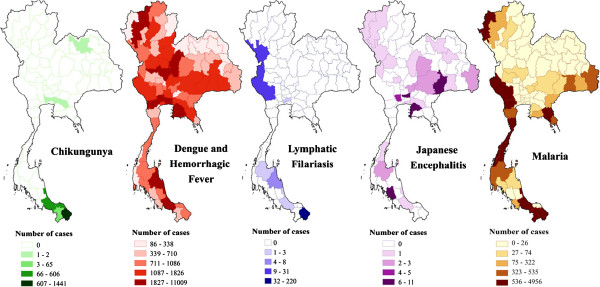
Distribution of the principle mosquito-borne diseases in Thailand (2012).

Thailand continues to face endemic transmission and the re-emergence of mosquito-borne diseases, principally malaria, dengue fever and dengue hemorrhagic fever (DF/DHF), lymphatic filariasis, Japanese encephalitis and more recently Chikungunya virus [36]. All these parasites and viruses are transmitted to humans by suitable vector mosquito species, some of which are capable of transmitting more than one disease pathogen [38]. The current distribution of these diseases in Thailand is presented in Figure 2. Like other countries in this region, malaria displays significant geographical heterogeneity and is exemplified by more intensified “border malaria”, with most of the malaria cases concentrated along the Thai borders with Myanmar and Cambodia [7,39-41], and more recently with the upsurge of malaria near the border with northern Malaysia [36]. According to [42], 8% of the total Thai population (~ 5 million inhabitants) resides in high risk areas for malaria (i.e., 1 case per 1000 population), 42% occupy lower risk areas (29 million population), while 50% are free from exposure to active malaria transmission (34 millions). The predominant malaria parasite species are *Plasmodium falciparum* and *Plasmodium vivax* but *P. vivax* has become slightly more prevalent than *P. falciparum* since 2000 [43].

Dengue viruses (*Flavivirus*) are responsible for one of the most prevalent vector-borne disease entities (DF/DHF) of public health importance in much of the tropical and subtropical world [44]. Each year, between 50–100 million dengue infections occur and nearly half the world population lives in the countries where dengue transmission is now endemic [45]. Much of urban Thailand is regarded as hyper-endemic for dengue infection with all four serotypes (DEN-1, DEN-2, DEN-3, DEN-4) regularly circulating and now occurring more commonly in rural areas. Unlike the majority of countries in the Southeast Asian Region, the total dengue cases reported in 2011 (78,337), 2010 (68,386) and 2009 (115,768) greatly exceeded that recorded in 2008 (56,651) with an increase in the proportion of severe dengue cases being reported [46]. Chikungunya (an *Alphavirus*) is another arboviral disease, very similar in epidemiology and clinical presentation to dengue, but occupying different antigenic families, Togaviridae vs. Flaviviridae, respectively. Compared to dengue, chikungunya is a relatively uncommon reported disease and likely misdiagnosed as classic dengue infection where they co-exist [47,48]. Although periodic outbreaks occur throughout Africa and Southeast Asia, they are typically self-limiting infections and rarely result in severe disease. After years of apparent quiescence, chikungunya emerged in 2004 in Kenya and subsequently invaded the Indian Ocean islands of Comoros and Réunion in 2005 and rapidly spread to other islands of the Indian Ocean reaching India in 2006 where more than 1 million suspected cases were reported [48]. In the succeeding years, Sri Lanka, Indonesia, Singapore and Malaysia were affected, including reports of significant outbreaks of chikungunya in the south of Thailand in which 49,069 infections were reported with a notable increase of neurologic complications [36,49].

Japanese encephalitis (JE), a *Flavivirus*, is the leading cause of viral encephalitis in Asian countries with approximately 30,000–50,000 cases reported annually, with case fatality rates between 0.3% and 60%, depending on the age at time of infection and severity of symptoms [50]. In SE Asia, JE is rarely reported among travelers to countries where JE is endemic [51]. The number of overt cases has shown a sharp decline after mass immunization programs in the region. Transmission appears to be declining mostly in China, Japan, and Korea, but cases continue to be reported from Bangladesh, Myanmar, India, Nepal, Sri Lanka, Vietnam and Thailand. Japanese encephalitis was first recognized in Japan in the 1920s and is believed to have spread from India throughout Asia, Indonesia and more recently northern Australia and New Guinea Island [52,53]. Typically, JE occurs as outbreaks in the extensive rice growing areas of Asia associated with *Culex tritaeniorhynchus, Culex gelidus,* and *Culex vishnui* group mosquitoes [54,55], especially in Thailand [36]. Even through developing symptomatic illnesses, humans, cattle and horses are regarded as dead-end hosts and not involved in the natural transmission cycle that involves a bird-mosquito maintenance cycle and various species of aquatic wading birds (e.g., ardeid birds) and pigs as principal amplifying hosts.

Lymphatic filariasis (LF) is caused by several species of nematodes that reside in the lymphatic system of the vertebrate host and is estimated to collectively infect more than 120 million people worldwide [56,57]. This disease is known for its debilitating and disfiguring outcome in the more unfortunate, albeit relatively rare, cases. Filariasis remains a major public health threat in many Southeast Asian countries where it is endemic in 9 of 11 countries. In Thailand, both *Wuchereria bancrofti* and *Brugia malayi* are presently and widely distributed, particularly along the international borders. *Wuchereria bancrofti* has been found along the western Thai-Myanmar border, including the provinces of Ranong, Ratchaburi, Kanchanaburi, Tak and Mae Hongsorn [58]. The most common filarial parasite strain in Thailand is the nocturnal sub-periodic form with a distinct peak of circulating microfilaria in the peripheral blood occurring between 18.00 and 20.00 h [59,60].

### Malaria vectors

Of the approximately 73 *Anopheles* species found in Thailand, select members of the Leucosphyrus Group (Neomyzomyia Series), Maculatus Group (Neocellia Series), and Minimus Subgroup (Myzomyia Series), as the most important malaria vectors in the country [38]. Five species within these 3 assemblages are incriminated as primary malaria vectors in Thailand, including *Anopheles baimaii* (previously *An. dirus* D) [61], *Anopheles dirus*[61,62], *Anopheles minimus* (previously species A) [63], *Anopheles pseudowillmori*[61] and *Anopheles aconitus*[61,64,65]. Manguin *et al*. [38] provides a current review of the vectorial capacity and bionomics of malaria vectors in the SEA region.

Several other potential vectors of malaria in Thailand that have a close association with humans, included *An. maculatus*, *Anopheles epiroticus* (= *An. sundaicus* A), *Anopheles karwari, Anopheles philippinensis,* and *Anopheles tessellatus*. Additionally, some members of the Barbirostris Group, subgenus *Anopheles* (*Anopheles barbirostris* and *Anopheles campestris*) exhibit malaria vector potential in Thailand [66].

### Dengue vectors

Only two species of *Aedes* mosquitoes, *Aedes aegypti* and *Aedes albopictus* are considered as primary vectors of dengue viruses in Thailand [67]. *Aedes aegypti* is highly anthropophilic and often propagates in and around human dwellings with a high propensity for resting inside houses. Larval habitats are typically artificial containers holding fresh water such as discarded tires, flower pots, drums, refuse bottles and cans, and other water storage devices [67,68]. *Aedes albopictus* is believed to be a native to Southeast Asia [69]. *Aedes albopictus* prefers to breed in natural habitats like tree holes, bamboo stumps and other natural containers but will also utilize outdoor man-made habitats that typically contain a higher amount of organic matter than tolerated by *Ae. aegypti.* Predominantly a 'rural’ species in Thailand, *Ae. albopictus* has been reported invading residential areas of larger urban zones, especially in the vicinity of Bangkok [70].

Female *Ae. albopictus* also displays a greater preference to rest and feed outdoors [71,72]. Similar to *Ae. aegypti*, it is a daytime feeder and can be found resting in shady areas in shrubs nearer ground level [73]. Likewise, *Ae. albopictus* blood feeding activity peaks in the early morning and late afternoon and is considered an aggressive biter with a wider host feeding range (human, domestic and wild animals) than *Ae. aegypti*[74]. This species is also known to be a competent vector of several other viral human pathogens, including Chikungunya virus [75,76] and Eastern equine encephalitis virus [77].

### Lymphatic filariasis vectors

In Asia, at least 36 mosquito species belonging to six genera have been incriminated as either primary or secondary vectors of *W. bancrofti*, with the majority being *Anopheles* species (24) followed by aedine mosquitoes (7 spp.), *Culex* (4 spp.) and two *Mansonia* (*M. dives*, *M. uniformis*) [38]. In Thailand, two species of *Mansonia* and five *Anopheles* are vectors of *Brugia malayi*[78]. Larval habitats for many of these vector species are commonly marshes/swamps found in close proximity to villages. The NSP form of the parasite has been experimentally and naturally transmitted by several mosquito genera, including *Aedes*, *Mansonia*, *Anopheles* and *Downsiomyia*[58,60]. In Thailand, lymphatic filariasis and malaria parasites can naturally share the same vector species, in particular *Anopheles dirus* and *Anopheles minimus* complexes, the *Anopheles maculatus* group, *Anopheles aconitus* and *Anopheles vagus*[38]. Active cases of filariasis in Thailand have declined over the past 20 years. Transmission is now found primarily with at-risk Thai migrants who enter endemic areas where vectors are common, especially along the Thai-Myanmar border. Increases in temporary migrant workers along the border have been associated with increases in disease transmission in the region. The prevalence of filariasis among a group of migrant workers in Tak Province, western Thailand, was approximately 4.4% and 2.4% in Prachuab Kiri Khan Province, southern Thailand [79,80].

### Japanese encephalitis vectors

In Thailand, JE virus is maintained enzootically within the rice field–breeding mosquitoes *Cx. tritaeniorhynchus*, *Cx. fuscocephala*, and *Cx. gelidus*[81]. Burke and Leake [82] have also reported *Cx. pseudovishnui* and *Cx. vishnui* to be competent vectors of JE and more recently, the virus has also been isolated from *Culex quinquefasciatus* in Thailand [83]. These mosquitoes can serve as the 'bridging’ vectors to humans from pigs that are the primary amplifying hosts in endemic areas and native wading birds associated with rice fields and natural wetlands as the natural virus reservoirs [84].

### Chikungunya vectors

The virus can be transmitted to humans via several species of mosquitoes, most notably several *Aedes* species, but also *Culex annulirostris*, *Mansonia uniformis* and a few species of *Anopheles*[85-87]. In Asia, the same two species that transmit dengue viruses, *Ae. aegypti* and *Ae. albopictus*, are of prime importance in chikungunya transmission in both urban and rural settings [88,89]. *Aedes albopictus* has been shown to have a higher susceptibility and a greater propensity to transmit the chikungunya virus than *Ae. aegypti*[75,76,90,91]. Similar to *Ae. aegypti*, the normal flight range of *Ae. albopictus* is generally limited within a radius of 400–600 m from their original larval habitat [92]. Like dengue, the virus has also been shown to be vertically transmitted from an infected female mosquito to her eggs and prodigy. The rapid extension of strains of *Ae. albopictus* worldwide, especially in temperate areas, represents a serious potential threat of chikungunya transmission in areas where it has not been seen before.

### Physiological resistance to insecticides

One of the primary methods of preventing vector-borne disease transmission is to disrupt human-vector contact using chemical means [12,13]. Synthetic chemicals of various classes have been used for many years in national public health vector control programs [1,2], currently the majority of which are pyrethroid-based formulations. In Thailand, combinations of different pyrethroids are commercially available to home owners to control mosquitoes and other indoor/outdoor arthropod pests. Pyrethroids have become relatively inexpensive, provide quick knockdown and are relatively safe compounds to use near humans to control common house-frequenting mosquitoes [8,93]. Since 1994, deltamethrin has been intensively used in organized vector control programs in Thailand to interrupt dengue transmission, generally in response to an outbreak. Numerous pyrethroid-based formulations (e.g., aerosols, coils, spray and gels), that include one or more of 12 different active ingredients, are available commercially to the general public [8,35,94]. A survey of 23 household products in public markets in metropolitan Bangkok found 11 containing varying low concentrations of permethrin, 11 with tetramethrin, 4 with bioallethrin and prallethrin, many with a synergist (piperonyl butoxide) added. Only three products had a mixture of deltamethrin (Table 1). One carbamate (propoxur) and one organophosphate (dichlorvos) are also available as an approved mixture in household pest control products. Frequent exposure of mosquito populations to sub-lethal concentrations of these chemicals may result in, or contribute to, the development of insecticide resistance with a direct operational impact on the effective management and prevention of vector-borne diseases [95].

In Thailand, resistance to DDT has been documented in only 2 species of anophelines (*Anopheles annularis* and *Anopheles minimus*) (Table 2), located in the northwestern part of the country [96]. In spite of decades of organized control activities using chemicals against malaria vectors, particularly IRS, there has been no published data reporting resistance to any other chemical class or active ingredient (mainly deltamethrin) used for vector control. This interesting finding contrasts significantly with the resistance patterns seen in *Ae. aegypti*, *Ae. albopictus* and *Cx. quinquefasciatus* in the country and could be explained by the predominant exophilic/exophagic behavior of major malaria vectors in the region that would limit the exposure time of mosquitoes with residual insecticides present inside houses (e.g., IRS and bednets).

**Table 2:** **List of*Anopheles*populations resistant to DDT in Thailand using the WHO standard contact assay (2000–2010)**

**Species**	**Insecticide**	**Location (province-district)**	**Geographic coordinates (DMS)**	**Published sources**
*Anopheles annularis*	DDT	Chiang Mai-Chiang Dao	19°32'N 98°54'E	Prapanthadara *et al*. 2000 [96]
*Anopheles annularis*	DDT	Mae Hongsorn	19°9'N 98°1'E	
*Anopheles minimus* (A)*	DDT	Phrae	18°6'N 100°16'E	

*An. minimus sensu strict. Note that resistance to DDT was also seen in non-vector anophelines of Thailand *before* 2000, *Anopheles aconitus*, *Anopheles culicifacies*, *Anopheles nivipes* and *Anopheles philippinensis*[8].

Resistance or incipient (tolerance) resistance to temephos, a common organophosphate used to control mosquito larvae, has been observed in *Ae. aegypti* in Thailand [13,97,98]. Of 19 populations tested, 12 were found resistant to temephos while 7 populations demonstrated tolerance. The degree of susceptibility to temephos also appeared to vary depending upon the history of chemical usage/exposure in the area. Data on the susceptibility to malathion and fenitrothion (organophosphates) and propoxur (carbamate) in adult *Ae. aegypti* has been compiled from 2000–2011 based on WHO susceptibility tests (Table 3). The degree of susceptibility to these two chemical groups also varied according to the geographic settings and previous chemical exposure. For example, strong resistance to malathion and fenitrothion in *Ae. aegypti* has been reported in the central, northern and northeastern areas of Thailand; whereas only moderate to low grades of resistance have been seen in the far north of the country [13,97]. Only one locality in southern Thailand has reported significant malathion resistance in *Ae. aegypti* populations [14].

**Table 3:** **Locations in Thailand with*Aedes aegypti*populations tested against insecticides using the WHO standard contact assay (2000–2011)**

**Insecticides**	**Location (province-district)**	**Geographic coordinates (DMS)**	**Published sources**
Cyfluthrin	Nonthaburi-Mueang	13°51′44″N 100°30′48″E	Paeporn *et al*. 2010 [99]
	Saraburi-Mueang *	14°31′38″N 100°54′35″E	
	Singburi-Mueang	14°53′18″N 100°24′17″E	
	Phitsanulok-Mueang	16°49′29″N 100°15′34″E	
	Phichit-Mueang	16°26′18″N 100°21′0″E	
	Sukhothai-Mueang	17°0′28″N 99°49′23″E	
	Uttaradit-Mueang	17°37′33″N 100°5′48″E	
	Lamphun-Mueang	18°34′42″N 99°1′6″E	
	Chiang Mai-Mueang	18°47′25″N 98°59′4″E	
	Chiang Rai-Mueang	19°54′31″N 99°49′57″E	
	Khon Kean-Mueang	16°26′18″N 102°50′20″E	
	Prachinburi-Mueang	14°3′2″N 101°22′0″E	
	Sra Kaeo-Mueang	13°48′52″N 102°4′20″E	Satimai 2010 [100]
	Rayong-Mueang	12°43′3″N 101°23′31″E	
	Chanthaburi-Mueang	12°36′40″N 102°6′15″E	
	Trat-Mueang *	12°13′54″N 102°30′48″E	
Cypermethrin	Chiang Mai-Mae Tang	19°10′N 98°54′E	Chareonviriyaphap *et al*. 2006 [101]
	Kanchanaburi-Sai Yok	14°17′N 99°11′E	
DDT	Chiang Mai-Mae Tang	19°8′N 98°51′E	Prapanthadara *et al.* 2002 [102]
	Chiang Mai-Mae Tang	19°11′N 98°54′E	Somboon *et al*. 2003 [93]
	Chiang Mai-Mueang	18°46′N 98°57′E	
	Lampang-Mueang	18°23′N 99°31′E	
	Nan-Mueang	18°47′N 100°43′E	
	Chiang Mai-Mae Tang	19°9′N 98°47′E	Lumjuan *et al.* 2005 [103]
	Chiang Mai- Mae Tang	19°8′N 98°51′E	Prapanthadara *et al*. 2005 [104]
	Bangkok-Bang Khen	13°52′N 100°35′E	Yaicharoen *et al.* 2005 [105]
	Chiang Mai-Mae Tang	19°9′N 98°52′E	Sathantriphop *et al*. 2006 [106]
	Chonburi	13°22′0″N 100°58′60″E	Rajatileka *et al*. 2008 [107]
	Phang Nga	8°28′0″N 98°31′60″E	
	Phang Nga-Thap Pud	8°31′0″N 98°37′60″E	
	Chiang Mai-Mae Tang	19°14′N 98°59′E	Thanispong *et al*. 2008 [14]
	Pathum Thani-Lad Lumkeaw	14°02′N 100°24′E	
	Chiang Mai-Mueang	18°47′N 99°00′E	
	Kanchanaburi-Sai Yok	14°20′N 98°59′E	
	Nonthaburi-Mueang	13°53′N 100°29′E	
	Songkhla-Mueang	7°11′N 100°35′E	
	Satun-Mueang	6°37′N 100°03′E	
	Bangkok-Chatuchak	13°50′N 100°34′E	
	Lampang-Mueang	18°17′N 99°29′E	
	Tak-Mae Sot	16°46′N 98°34′E	
	Khon Kean-Mueang	16°25′N 102°50′E	
	Surat Thani-Mueang	9°08′N 99°20′E	
	Nakhon Sawan-Mueang	15°42′N 100°08′E	
	Kamphaeng Phet	16°28′0"N 99°30′0"E	
	Phang Nga-Takua Pa	8°52′0"N 98°20′60"E	
	Phuket	7°52′60″N 98°24′0″E	
Deltamethrin	Chiang Mai-Mueang	18°46′N 98°57′E	Somboon *et al*. 2003 [93]
	Nan-Muang	18°47′N 100°43′E	
	Ratchaburi-Pongsawai	13°32′43″N 99°51′7″E	Paeporn *et al*. 2004 [34]
	Ratchaburi-KhuBua	13°28′53″N 99°49′21″E	
	Chiang Mai-Mae Tang	19°10′N 98°54′E	Chareonviriyaphap *et al*. 2006 [101]
	Bangkok-Bang Khen	13°52′26″N 100°35′47″E	Yaicharoen *et al*. 2005 [105]
	Bangkok-Hauykwang	13°4′47.4″N 100°3′ 52.3″E	Jirakanjanakit *et al*. 2007 [13]
	Bangkok-Laksi	13°5′28.2″N 100° 3′ 41.2″E	
	Bangkok-Ladkrabang	13°4′47.5″N 100°4′23.6″E	
	Bangkok-Rasburana	13°3′59.2″N 100°3′58.8″E	
	Chonburi-Panusnikom	13°2′2.9″N 101°1′5.3″E	
	Chonburi-Banglamung	12°5′42.2″N 100°5′32.2″E	
	Nakhon Sawan-Taklee	15°1′53″N 100°1′48.2″E	
	Chanthaburi-Mueang	13°19′N 100°55′E	
	Nakhon Ratchasima-Kornburi	14°3′50″N 102°1′45.5″E	
	Nakhon Ratchasima-Senagsang	14°2′35.2″N 102°2′45.6″	
	Nakhon Sawan-Mae Pern	15°3′21″N 99°2′48″E	
	Nakhon Sawan-Mae Wong	15°4′35″N 99°3′7.4″E	
	Nakhon Ratchasrima-Kornburi	14°31′24″N 102°14′54″E	
	Bangkok-Hauykwang	13°45′54″N 100°34′39″E	Pethuan *et al*. 2007 [108]
	Bangkok-Laksi	13°53′15″N 100°34′44″E	
	Chonburi-Panusnikom	13°27′6″N 101°10′36″E	
	Chonburi-Banglamung	12°58′36″N 100°54′48″E	
	Nakhon Sawan-Taklee	15°15′47″N 100°20′37″E	
	Nakhon Ratchasrima-Serngsang	14°25′34″N 102°27′38″E	
	Nakhon Sawan-Mae Pern	15°39′28″N 99°28′9″E	
	Nakhon Sawan-Mae Wong	15°46′52″N 99°31′9″E	
	Chanthaburi-Makham *	12°40′25″N 102°11′48″E	Narksuwan *et al.* 2008 [109]
	Phetchaburi-Tha Yang *	12°58′24″N 99°53′16″E	
	Buri Ram-Cham ni *	14°47′18″N 102°50′30″E	
	Loei-Nong Hin *	17°7′24″N 101°51′30″E	
	Trang-Sikao *	07°34′18″N 99°20′42″E	
	Chum phon-Lang Suan	09°56′42″N 99°4′42″E	
	Phetchabun-Wichian Buri *	15°39′26″N 101°6′24″E	
	Kalasin-Yangtalat *	16°24′8″N 103°22′23″E	
	Lampang-Thoen *	17°36′42″N 99°12′57″E	
	Uthai Thani-Thap Than*	15°29′12″N 99°49′54″E	
	Suphanburi-Bang plama *	14°24′8″N 100°9′16″E	
	Ratchaburi-Damneon Saduak	13°31′6″N 99°57′18″E	
	Ayutthaya-Phak Hai *	14°27′30″N 100°22′12″E	
	Nonthaburi-Mueang	13°51′44″N 100°30′48″E	Paeporn *et al.* 2010 [99]
	Nakhon Pathom-Mueang	13°49′11″N 100°3′57″E	
	Sra Kaeo-Mueang *	13°48′53″N 102°4′19″E	
	Saraburi-Mueang *	14°31′38″N 100°54′35″E	
	Lopburi-Mueang	14°47′53″N 100°39′13″E	
	Suphanburi-Mueang	14°29′4″N 100°7′25″E	
	Angthong-Mueang	14°35′19″N 100°27′12″E	
	Singburi-Mueang	14°53′18″N 100°24′17″E	
	Kanchanaburi-Mueang	14°0′12″N 99°33′0″E	
	Phitsanulok-Mueang	16°49′29″N 100°15′34″E	
	Phichit-Mueang	16°26′18″N 100°21′0″E	
	Sukhothai-Mueang	17°0′28″N 99°49′23″E	
	Uttaradit-Mueang	17°37′33″N 100°5′48″E	
	Lamphun-Mueang	18°34′42″N 99°1′6″E	
	Uthaithani-Mueang *	15°22′46″N 100°1′29″E	
	Chiang Mai-Mueang	18°47′25″N 98°59′4″E	
	Chiang Rai-Mueang *	19°54′31″N 99°49′57″E	
	Khon Kean-Mueang	16°26′18″N 102°50′20″E	
	Udonthani-Mueang	17°24′54″N 102°47′12″E	
	Chai yaphum-Mueang	15°48′35″N 102°1′13″E	
	Nakhon Nayok-Mueang*	14°12′13″N 101°13′2″E	
	Rayong-Mueang *	12°40′6″N 101°16′30″E	
	Prachinburi-Mueang	14°3′2″N 101°22′0″E	
	Chonburi-Mueang	13°21′43″N 100°58′45″E	
	Sra Kaeo-Mueang *	13°48′52″N 102°4′20″E	Satimai 2010 [100]
	Rayong-Mueang	12°43′3″N 101°23′31″E	
	Chanthaburi-Mueang	12°36′40″N 102°6′15″E	
	Trat-Mueang *	12°13′54″N 102°30′48″E	
	Bangkok-Kannayaw	13°50′N 100°40′E	Chuaycharoensuk *et al*. 2011 [35]
	Chanthaburi-Mueang	12°39′N 102°7′E	
	Chonburi-Mueang	13°19′N 100°55′E	
	Khon Kean-Mueang	16°19′N 102°47′E	
	Udonthani-Wungsammor	15°54′N 103°28′E	
	Nakhon Sawan-Mueang	15°40′N 100°05′E	
	Tak-Mae Sot	16°43′N 98°34′E	
	Chumphon-Mueang	10°30′N 99°07′E	
	Prachuap Khiri Khan-Hua Hin	12°33′N 99°53′E	
	Songkhla-Namom	06°54′N 100°32′E	
	Songkhla-Sadao	06°45′N 100°24′E	
	Songkhla-Had Yai	07°00′N 100°27′E	
	Surat Thani-Mueang	09°02′N 99°22′E	
Etofenprox	Nan-Tha Wang Pha	19°7′N 100°43′E	Somboon *et al*. 2003 [93]
Fenitrothion	Nakhon Sawan-Mueang	15°4′47″N 100°34.7″E	Jirakanjanakit *et al*. 2007 [13]
	Nakhon Sawan-Krok Pra	15°3′12″N 100°33.7″E	
	Nakhon Ratchasima-Prathai	15°3′56″N 102°3′45.7″E	
	Nakhon Ratchasima-Kagsanamnang	15°4′14″N 102°1′36.9″E	
	Nakhon Ratchasima-Seekhew	14°5′19.5″N 101°4′28.8″E	
	Nakhon Ratchasima-Senagsang	14°2′35.2″N 102°2′45.6″E	
	Nakhon Sawan-Mae Pern	15°3′21″N 99°2′48″E	
	Nakhon Sawan-Mae Wong	15°4′35″N 99°3′7.4″E	
	Nakhon Ratchasrima-Seekhew	14°53′30″N 101°43′24″E	Pethuan *et al*. 2007 [108]
	Nakhon Ratchasrima-Prathai	15°32′0″N 102°43′22″E	
	Nakhon Ratchasrima-Kangsanamnang	15°45′0″N 102°15′17″E	
	Nakhon Sawan-Mueang	15°44′15″N 100°5′21″E	
	Nakhon Sawan-Krok Pra	15°34′48″N 100°0′13″E	
	Nakhon Ratchasrima-Serngsang	14°25′34″N 102°27′38″E	
	Nakhon Sawan-Mae Pern	15°39′28″N 99°28′9″E	
	Nakhon Sawan-Mae Wong	15°46′52″N 99°31′9″E	
	Nonthaburi -Mueang	13°51′44″N 100°30′48″E	Paeporn *et al*. 2010 [99]
	Sra Kaeo-Mueang *	13°48′53″N 102°4′19″E	
	Saraburi-Mueang *	14°31′38″N 100°54′35″E	
	Lopburi-Mueang	14°47′53″N 100°39′13″	
	Suphanburi-Mueang	14°29′4″N 100°7′25″E	
	Angthong-Mueang	14°35′19″N 100°27′12″E	
	Singburi-Mueang *	14°53′18″N 100°24′17″E	
	Phitsanulok-Mueang *	16°49′29″N 100°15′34″E	
	Phichit-Mueang	16°26′18″N 100°21′0″E	
	Sukhothai-Mueang	17°0′28″N 99°49′23″E	
	Uttaradit-Mueang*	17°37′33″N 100°5′48″E	
	Chiang Mai-Mueang	18°47′25″N 98°59′4″E	
	Khon Kean-Mueang	16°26′18″N 102°50′20″E	
	Udonthani-Mueang *	17°24′54″N 102°47′12″E	
	Rayong-Mueang*	12°40′6″N 101°16′30″E	
Lambdacyhalothrin	Sra Kaeo-Mueang	13°48′52″N 102°4′20″E	Satimai 2010 [100]
	Rayong-Mueang	12°43′3″N 101°23′31″E	
	Chanthaburi-Mueang	12°36′40″N 102°6′15″E	
	Trat-Mueang	12°13′54″N 102°30′48″E	
Malathion	Tak-Mae Sot	16°46′N 98°34′E	Thanispong *et al*. 2008 [14]
	Khon Kean-Mueang	16°25′N 102°50′E	
	Surat Thani-Mueang	9°08′N 99°20′E	
	Nakhon Sawan-Mueang	15°42′N 100°08′E	
	Nonthaburi-Mueang *	13°51′44″N 100°30′48″E	Paeporn *et al*. 2010 [99]
	Sra Kaeo-Mueang	13°48′53″N 102°4′19″E	
	Lopburi-Mueang	14°47′53″N 100°39′13″E	
	Suphanburi -Mueang *	14°29′4″N 100°7′25″E	
	Angthong-Mueang	14°35′19″N 100°27′12″E	
	Singburi-Mueang	14°53′18″N 100°24′17″E	
	Phichit-Mueang	16°26′18″N 100°21′0″E	
	Sukhothai-Mueang *	17°0′28″N 99°49′23″E	
	Uttaradit-Mueang *	17°37′33″N 100°5′48″E	
	Uthaithani-Mueang	15°22′46″N 100°1′29″E	
	Khon Kean-Mueang *	16°26′18″N 102°50′20″E	
	Udonthani-Mueang *	17°24′54″N 102°47′12″E	
Permethrin	Chiang Mai- Mae Tang	19°8′N 98°51′E	Prapanthadara *et al.* 2002 [102]
	Chiang Mai-Mueang	18°46′N 98°57′E	Somboon *et al.* 2003 [93]
	Nan-Mueang	18°47′N 100°43′E	
	Lampang-Mueang	19°11′N 98°54′E	
	Ratchaburi- Pongsawai	13°32′43″N 99°51′7″E	Paeporn *et al*. 2004 [34]
	Ratchaburi- KhuBua	13°28′53″N 99°49′21″E	
	Chiang Mai-Mae Tang	19°9′N 98°47′E	Lumjuan *et al*. 2005 [103]
	Tak-Mae Pa	16°45′N 98°33′E	Ponlawat *et al*. 2005 [98]
	Tak-Mae Pa	16°45′N 98°34′E	
	Nakhon Sawan-Phayuhakhiri	15°29′N 100°8′E	
	Surat Thani-Tha Chana	9°34′N 99°07′E	
	Phatthalung-Mueang	7°30′N 100°03′E	
	Nakhon Ratchasima-Kham Thale So	15°05′N 101°54′E	
	Chiang Mai- Mae Tang	19°8′N 98°51′E	Prapanthadara *et al.* 2005 [104]
	Chiang Mai-Mae Tang	19°9′N 98°52′E	Sathantriphop *et al.* 2006 [106]
	Nonthaburi-Baan Suan	13°51′N 100°29′E	
	Bangkok-Bangkoknoi	13°45′40″N 100°2′1.9″E	Jirakanjanakit *et al.* 2007 [13]
	Chonburi-Sriracha	13°19″N 101°11.8″E	
	Songkhla-Mueang	07°1′41.8″N 100°3′54.6″E	
	Kanchanaburi-Tamaka	13°55′15″N 99°45′56″E	
	Bangkok-Hauykwang	13°4′47.4″N 100°3′52.3″E	
	Bangkok-Laksi	13°5′28.2″N 100°3′41.2″E	
	Bangkok-Ladkrabang	13°4′47.5″N 100°4′23.6″E	
	Bangkok-Rasburana	13°3′59.2″N 100°3′58.8″E	
	Bangkok-Panusnikom	13°2′2.9″N 101°1′5.3″E	
	Bangkok-Banglamung	12°5′42.2″N 100°5′32.2″E	
	Nakhon Sawan-Taklee	15°1′53″N 100°1′48.2″E	
	Nakhon Sawan-Muang	15°4′47″N 100°34.7″E	
	Nakhon Sawan-Krok Pra	15°3′12″N 100°33.7″E	
	Nakhon Ratchasima-Senagsang	14°2′35.2″N 102°2′45.6″E	
	Nakhon Sawan-Mae Pern	15°3′21″N 99°2′48″E	
	Nakhon Sawan-Mae Wong	15°4′35″N 99°3′7.4″E	
	Bangkok-Hauykwang	13°45′54″N 100°34′39″	Pethuan *et al.* 2007 [108]
	Bangkok-Laksi	13°53′15″N 100°34′44″E	
	Chonburi-Panusnikom	13°27′6″N 101°10′36″E	
	Chonburi-Banglamung	12°58′36″N 100°54′48″E	
	Nakhon Sawan	15°15′47″N 100°20′37″E	
	Nakhon Sawan-Mueang	15°44′15″N 100°5′21″E	
	Nakhon Sawan-Krok Pra	15°34′48″N 100°0′13″E	
	Nakhon Ratchasrima-Serngsang	14°25′34″N 102°27′38″E	
	Nakhon Sawan-Mae Pern	15°39′28″N 99°28′9″E	
	Nakhon Sawan-Mae Wong	15°46′52″N 99°31′9″E	
	Chonburi	13°22′0″N 100°58′60″E	Rajatileka *et al*. 2008 [107]
	Phang Nga	8°28′0″N 98°31′60″E	
	Phang Nga-Thap Pud	8°31′0″N 98°37′60″E	
	Chiang Mai-Mueang	18°47′N 99°00′E	Thanispong *et al.* 2008 [14]
	Kanchanaburi-Sai Yok	14°20′N 98°59′E	
	Nonthaburi-Mueang	13°53′N 100°29′E	
	Songkhla-Mueang	7°11′N 100°35′E	
	Satun-Mueang	6°37′N 100°03′E	
	Bangkok-Chatuchak	13°50′N 100°34′E	
	Lampang-Mueang	18°17′N 99°29′E	
	Tak-Mae Sot	16°46′N 98°34′E	
	Khon Kean-Mueang	16°25′N 102°50′E	
	Surat Thani-Mueang	9°08′N 99°20′E	
	Nakhon Sawan-Mueang	15°42′N 100°08′E	
	Nonthaburi-Mueang	13°51′44″N 100°30′48″E	Paeporn *et al*. 2010 [99]
	Nakhon Pathom-Mueang	13°49′11″N 100°3′57″	
	Sra Kaeo-Mueang	13°48′53″N 102°4′19″E	
	Saraburi-Mueang	14°31′38″N 100°54′35″E	
	Lopburi-Mueang	14°47′53″N 100°39′13″E	
	Suphanburi-Mueang	14°29′4″N 100°7′25″E	
	Angthong-Mueang	14°35′19″N 100°27′12″E	
	Singburi-Mueang	14°53′18″N 100°24′17″E	
	Kanchanaburi-Mueang	14°0′12″N 99°33′0″E	
	Phitsanulok-Mueang	16°49′29″N 100°15′34″E	
	Phichit-Mueang	16°26′18″N 100°21′0″E	
	Sukhothai-Mueang	17°0′28″N 99°49′23″E	
	Uttaradit-Mueang	17°37′33″N 100°5′48″E	
	Lamphun-Mueang	18°34′42″N 99°1′6″E	
	Uthaithani-Mueang	15°22′46″N 100°1′29″E	
	Chiang Mai-Mueang	18°47′25″N 98°59′4″E	
	Chiang Rai-Mueang	19°54′31″N 99°49′57″E	
	Khon Kean-Mueang	16°26′18″N 102°50′20″E	
	Udonthani-Mueang	17°24′54″N 102°47′12″E	
	Chai yaphum-Mueang	15°48′35″N 102°1′13″E	
	Nongkhai-Mueang	17°52′48″N 102°44′30″E	
	Nakhon Nayok-Mueang	14°12′13″N 101°13′2″E	
	Rayong-Mueang	12°40′6″N 101°16′30″E	
	Prachinburi-Mueang	14°3′2″N 101°22′0″E	
	Chonburi-Mueang	13°21′43″N 100°58′45″E	
	Sra Kaeo-Mueang	13°48′52″N 102°4′20″E	Satimai 2010 [100]
	Rayong-Mueang	12°43′3″N 101°23′31″E	
	Chanthaburi-Mueang	12°36′40″N 102°6′15″E	
	Trat-Mueang	12°13′54″N 102°30′48″E	
	Bangkok-Kannayaw	13°50′N 100°40′E	Chuaycharoensuk *et al.* 2011 [35]
	Chanthaburi-Mueang	12°39′N 102°7′E	
	Chonburi-Mueang	13°19′N 100°55′E	
	Prachinburi-Mueang	14°7′N 101°21′E	
	Buri Rum-Lam Plai Mat	15°00′N 102°50′E	
	Kalasin-Huaypung	16°40′N 103°53′E	
	Prachinburi-Mueang	14°6′N 101°19′E	
	Khon Kean-Mueang	16°19′N 102°47′E	
	Nakhon Ratchasima-WangNam Kheow	14°26′N 101°47′E	
	Srisaket-Uthumpronpisai	15°08′N 104°12′E	
	Surin-Dontoom	15°14′N 103°30′E	
	Roi Et-Suwannaphum	15°40′N 103°49′E	
	Nakhon Ratchasima-WangNam Kheow	14°24′N 101°51′E	
	Udonthani-Wungsammor	17°3′N 103°26′E	
	Chiang Mai-Mueang	18°46′N 98°56′E	
	Chiang Rai-Mae Chun	20°08′N 99°51′E	
	Kampaeng Phet-Kanuworralukburi	16°00′N 99°48′E	
	Lampang-Mueang	18°14′N 99°26′E	
	Lamphun-Mueang	18°37′N 99°00′E	
	Nakhon Sawan-Mueang	15°40′N 100°05′E	
	Phrae-Mueang	18°05′N 100°12′E	
	Tak-Mae Sot	16°43′N 98°34′E	
	Uthaithani-Ban Rai	15°12′N 99°41′E	
	Chumphon-Mueang	10°30′N 99° 07′E	
	Phang Nga-Takuaytung	08°12′N 98°17′E	
	Phatthalung-Pa Bon	07°16′N 100°09′E	
	Phuket-Mueang	07°53′N 98°23′E	
	Prachuap Khiri Khan-Hua Hin	12°33′N 99°53′E	
	Songkhla-Namom	06°54′N 100°32′E	
	Songkhla-Ranode	07°52′N 100°18′E	
	Songkhla-Sadao	06°45′N 100°24′E	
	Songkhla-Had Yai	07°00′N 100°27′E	
	Surat Thani-Mueang	09°02′N 99°22′E	
Propoxur	Nakhon Sawan-Mae Wong	15°4′35″N 99°3′7.4″E	Jirakanjanakit *et al.* 2007 [13]
	Nakhon Sawan-Mae Wong	15°46′52″N 99°31′9″E	Pethuan *et al*. 2007 [108]
	Surat Thani-Mueang	9°08′N 99°20′E	Thanispong *et al*. 2008 [14]
	Nakhon Sawan-Muang	15°42′N 100°08′E	
Temephos	Tak-Mae Pa	16°45′N 98°33′E	Ponlawat *et al*. 2005 [98]
	Tak-Mae Pa	16°45′N 98°34′E	
	Nakhon Sawan-Phayuhakhiri	15°29′N 100°8′E	
	Surat Thani-Tha Chana	9°34′N 99°07′E	
	Phatthalung-Mueang	7°30′N 100°03′E	
	Roi Et-Mueang	16°03′N 103°39′E	Saelim *et al*. 2005 [110]
	Bangkok- Hauykwang	13°4′47.4″N 100°3′52.3″E	Jirakanjanakit *et al.* 2007 [111]
	Nakhon Sawan-Mae Wong	15°4′35″N 99°3′7.4″E	
	Nakhon Ratchasrima- Prathai	15°3′56″N 102°3′45.7″E	
	Nonthaburi-Mueang *	13°51′44″N 100°30′48″E	Paeporn *et al*. 2010 [99]
	Suphanburi-Mueang *	14°29′4″N 100°7′25″E	
	Angthong-Mueang	14°35′19″N 100°27′12″E	
	Lopburi-Mueang	14°47′53″N 100°39′13″E	
	Chiang Mai-Mueang *	18°47′25″N 98°59′4″E	
	Uthaithani-Mueang	15°22′46″N 100°1′29″E	
	Nongkhai-Mueang *	17°52′48″N 102°44′30″E	
	Khon Kean-Mueang *	16°26′18″N 102°50′20″E	
	Chanthaburi-Mueang *	12°36′38″N 102°6′15″E	
	Chanthaburi-Mueang *	12°36′40″N 102°6′15″E	Satimai 2010 [100]

*Evidence of low-grade insecticide resistance.

**Figure 3:**
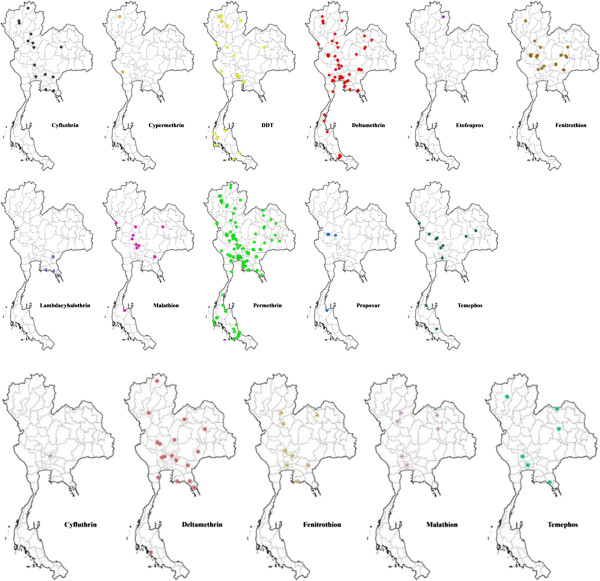
**Distribution of *Aedes aegypti *with resistance to insecticides in Thailand (2000–2011).**

**Figure 4:**
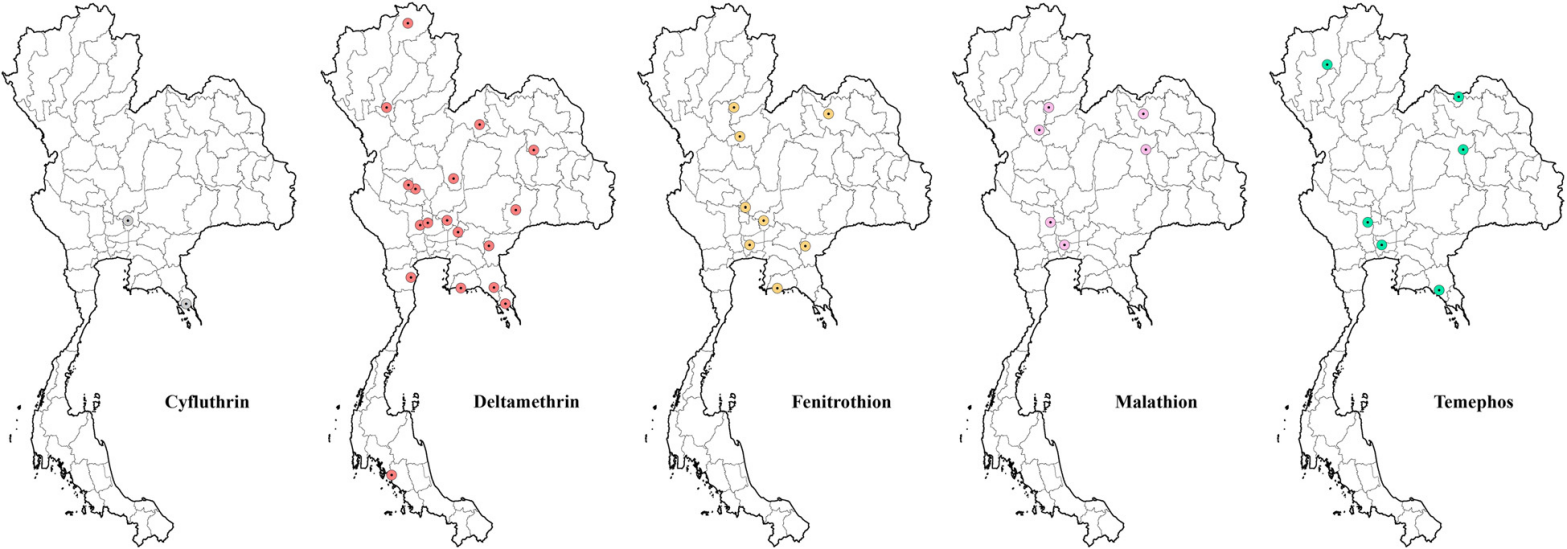
**Distribution of *Aedes aegypti *with resistance to incipient insecticides in Thailand (2000–2011).**

**Figure 5:**
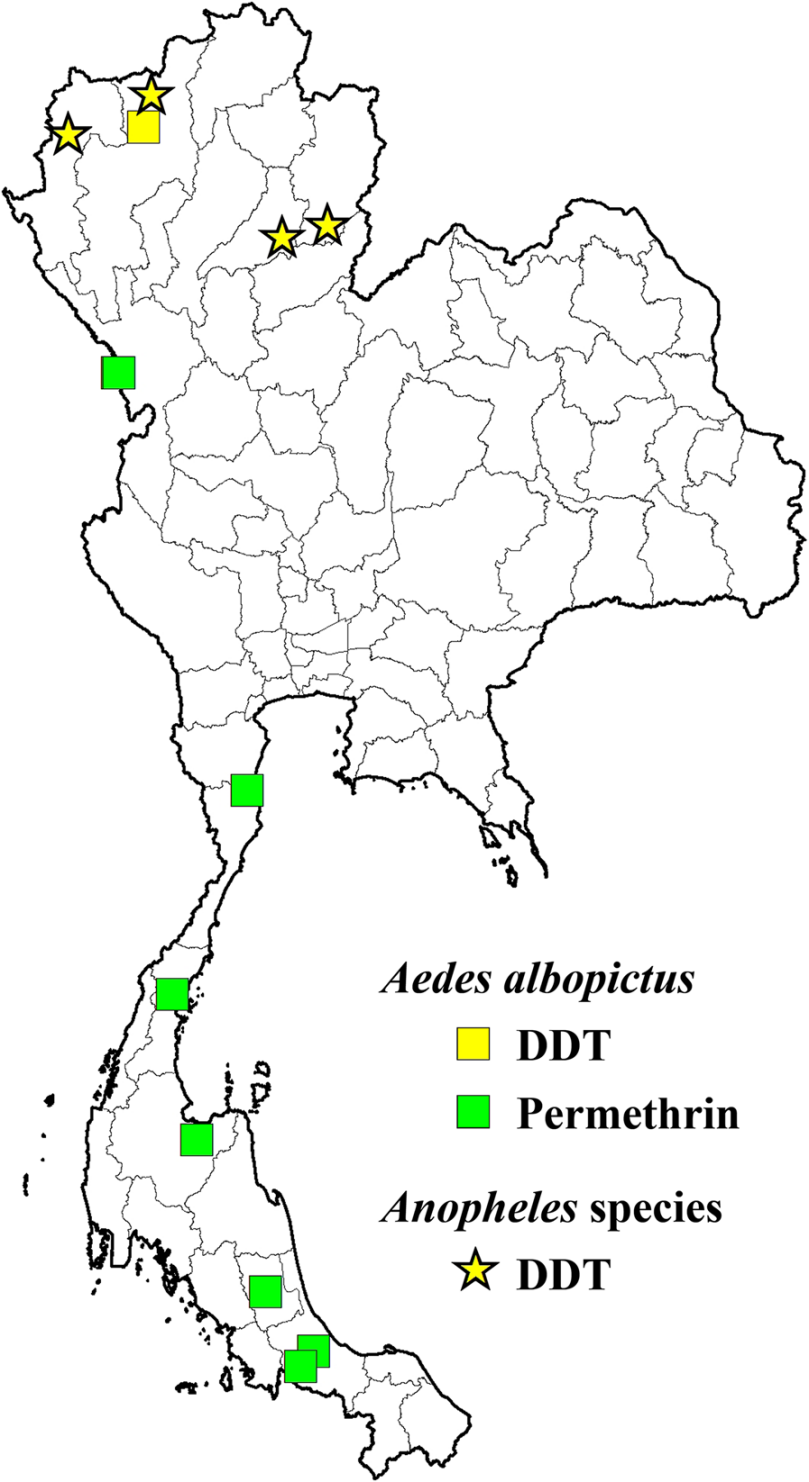
**Distribution of *Aedes albopictus *and *Anopheles *species with resistance to DDT and permethrin, and DDT, respectively, in Thailand (2000–2011).**

Thanispong *et al*. [14], reported *Ae. aegypti* resistant to six different synthetic pyrethroids, namely (cyfluthrin, cypermethrin, deltamethrin, etofenprox, lambda-cyhalothrin, and permethrin) (Table 3). Permethrin resistance is widely distributed in the country, while deltamethrin, lambda-cyhalothrin and cyfluthrin resistance has so far shown a much narrower spatial distribution (Figure 3). DDT resistance has been found throughout the country, whereas fenitrothion (OP) was restricted to the central and northern regions of Thailand (Figure 3). In addition, incipient resistance has been reported for three major groups of insecticides, with the majority being synthetic pyrethroids, especially permethrin and deltamethrin (Figure 4). Although data is limited, temephos resistance (OP) in *Ae*. *aegypti* appears more prevalent than resistance to propoxur (carbamate) (Figure 3). For *Aedes albopictus*, few reports on physiological resistance patterns are available; however, most of the *Ae. albopictus* populations sampled have demonstrated resistance to permethrin and one population showed resistance to DDT (Table 4 and Figure 5).

**Table 4:** **Locations in Thailand with*Aedes albopictus*populations tested against insecticides using the WHO standard contact assay (2000–2011)**

**Insecticide**	**Location (province-district)**	**Geographic coordinates (DMS)**	**Published sources**
DDT	Chiang Mai-Mae Tang	19°11′N 98°54′E	Somboon *et al*. 2003 [93]
Permethrin	Phatthalung-Mueang	7°30′N 100°03′E	Ponlawat *et al.* 2005 [98]
	Tak-Mae Sot	16°45′N 98°33′E	
	Tak-Mae Sot	16°45′N 98°34′E	
	Chumphon-Mueang	10°30′N 99°07′E	Chuaycharoensuk *et al.* 2011 [35]
	Prachuap Khiri Khan-Hua Hin	12°33′N 99°53′E	
	Songkhla-Namom	6°54′N 100°32′E	
	Songkhla-Sadao	6°45′N 100°24′E	
	Surat Thani-Mueang	9°02′N 99°22′E	

More recent data on pyrethroid resistance in *Ae. aegypti* and *Ae*. *albopictus* populations from Thailand have been restricted in specific geographical areas [8,13,14,34,105,106,112]. Physiological resistance to three different pyrethroids among 32 *Ae. aegypti* strains collected throughout Thailand and five strains of *Ae. albopictus* from southern Thailand were investigated [35]. The frequency of resistance to permethrin in *Ae. aegypti* populations varied between 4% and 56.4%. All 32 strains of *Ae. aegypti* were found to have evidence of incipient resistance (62.5%) or levels of permethrin post-exposure survival deemed clearly resistant (37.5%). However, the majority of *Ae. aegypti* strains were found to be susceptible (> 98% mortality) to deltamethrin. Four strains of *Ae. albopictus* showed evidence of incipient resistance to lambda-cyhalothrin and one showed high resistance to permethrin [35].

**Figure 6:**
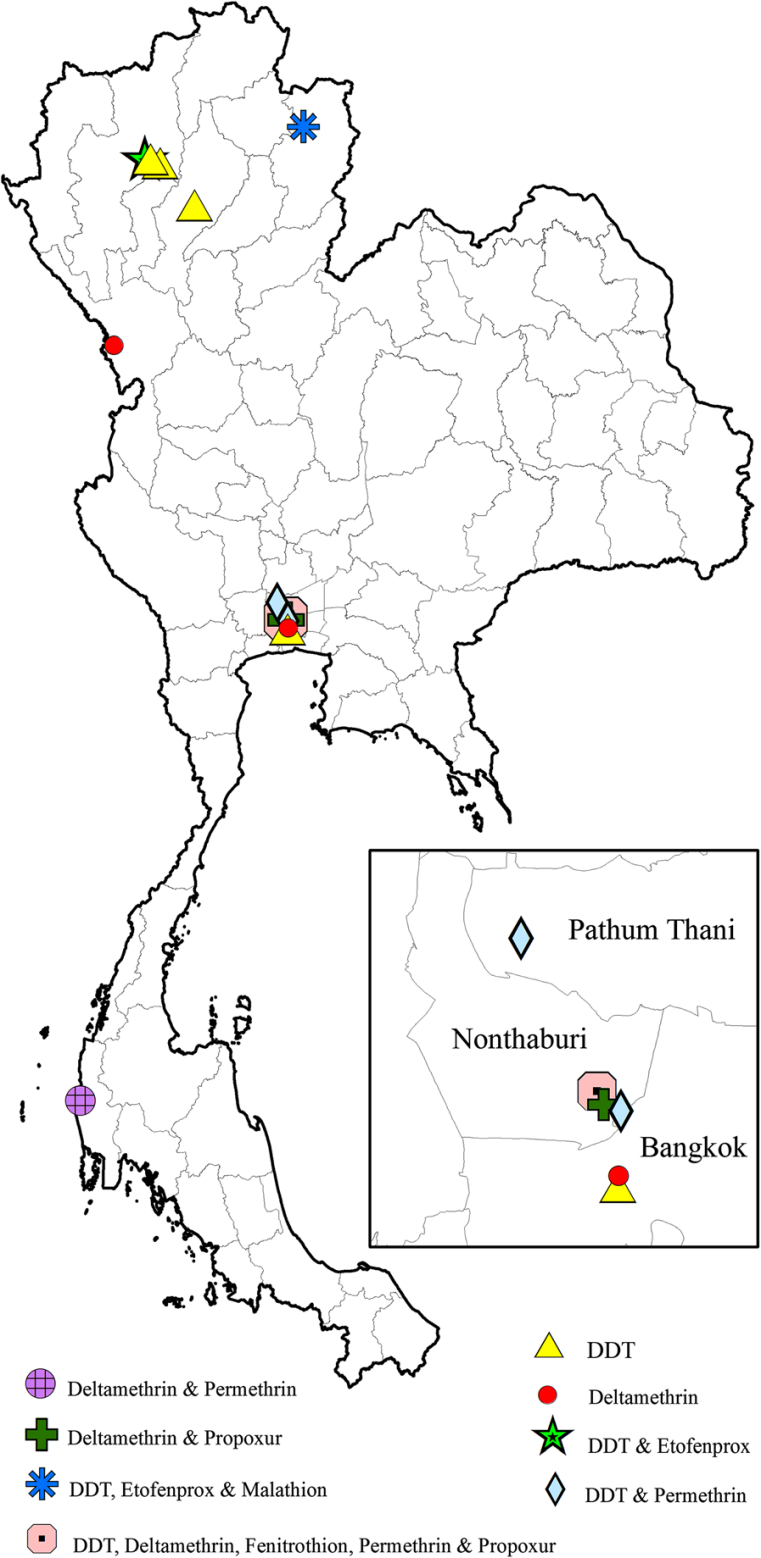
**Distribution of *Culex quinquefasciatus *with resistance to insecticides in Thailand (2000–2011).**

In *Cx. quinquefasciatus*, there were high levels of resistance to DDT, permethrin, deltamethrin and propoxur, whereas susceptibility to malathion and fenitrothion (Table 5, Figure 6) was maintained [93,113]. However, the limited number of sites and populations tested render it difficult to estimate the country-wide importance and impact of insecticide resistance in this mosquito species.

**Table 5:** **Locations in Thailand with*Culex quinquefasciatus*populations tested against insecticides using the WHO standard contact assay (2000–2011)**

**Insecticide**	**Location (province-district)**	**Geographic coordinates (DMS)**	**Sources**
DDT	Chiang Mai- Mueang	18°47′N 99°1′E	Prapanthadara *et al.* 2000 [96]
	Chiang Mai-Mueang	18°46′N 98°57′E	Somboon *et al*. 2003 [93]
	Chiang Mai-San Kampaeng	18°44′43″N 99°7′13″E	
	Lampang-Mueang	18°17′31″N 99°30′16″E	
	Nan-Tha Wang Pha	19°7′35″N 100°48′53″E	
	Nonthaburi-Baan Suan	13°51′N 100°29′E	Sathantriphop *et al.* 2006 [113]
	Bangkok-Phom Pabsatupai	13°44′N 100°29′E	Thanispong *et al.* 2008 [14]
	Nonthaburi-Mueang	13°50′N 100°31′E	
	Pathum Thani-Lad Lumkeaw	14°02′N 100°24′E	
Deltamethrin	Phang-Nga- Keuk-kak	8°41′47.2″N 98°15′28.6″E	Komalamisra *et al*. 2006 [114]
	Nonthaburi-Baan Suan	13°51′N 100°29′E	Sathantriphop *et al*. 2006 [106]
	Nonthaburi-Muenng	13°50′N 100°29″E	Sathantriphop *et al.* 2006 [113]
	Tak-Mae Sot	16°47′N 98°36′E	
	Bangkok-Pom Prab Satru Phai	13°45′N 100°30′E	
	(laboratory strain) in 1978		
Etofenprox	Chiang Mai-Mueang	13°19′N 100°55′E	Somboon *et al*. 2003 [93]
	Nan-Tha Wang Pha	19°7′35″N 100°48′53″E	
Fenitrothion	Nonthaburi-Baan Suan	13°51′N 100°29′E	Sathantriphop *et al.* 2006 [106]
Malathion	Nan-Tha Wang Pha	19°7′35″N 100°48′53″E	Somboon *et al.* 2003 [93]
Permethrin	Phang-Nga Keuk-kak	8°41′47.2″N 98°15′28.6″E	Komalamisra *et al.* 2006 [114]
	Nonthaburi-Baan Suan	13°51′N 100°29′E	Sathantriphop *et al.* 2006 [106]
	Nonthaburi-Mueang	13°50′N 100°31′E	Thanispong *et al*. 2008 [14]
	Pathum Thani-Lad Lumkeaw	14°02′N 100°24′E	
Propoxur	Nonthaburi-Baan Suan	13°51′N 100°29′E	Sathantriphop *et al*. 2006 [106]
	Nonthaburi-Mueang	13°50′30″N 100°29′45″E	Sathantriphop *et al.* 2006 [113]

### Behavioral responses to insecticides

Behavioral responses of mosquitoes to insecticides can be observed using various laboratory devices and field 'excito-repellency’ assay systems. For laboratory assays, many of the assay variations have been reviewed [12]. The World Health Organization developed the first test box to evaluate the excitation (“irritability”) of exposed mosquitoes following physical contact with insecticides [115]. This system was subsequently referred to as an “excito-repellency” test box [116]. The test system was further modified by other investigators interested in behavioral avoidance responses to DDT and some of the early synthetic pyrethroids [12,117-121]. A light-proof test chamber was designed to study the irritant response of *Anopheles gambiae*, an important malaria vector in Africa, to several chemical compounds [122]. One limitation associated with all of these earlier test systems was the procedural difficulty for introducing and removing test specimens with the assay designs. Other concerns were the ability to evaluate various physiological states found in wild-caught mosquitoes and selecting the ideal range of concentrations for chemical evaluation. Moreover, at the time no universal analysis or set of statistical methods for interpretation of data had been fully accepted nor had any test system been designed to more clearly discriminate between contact excitation and noncontact (spatial) repellency responses [12]. An improved excito-repellency test device that was able to better differentiate between excitation and spatial repellency was developed and first tested against field populations of *Anopheles albimanus* in Central America [12,19]. Unfortunately, this fixed prototype was cumbersome to handle and required considerable time for attaching the insecticide-treated test papers inside the chamber. Eventually, a more field deployable test system was designed that was collapsible and easily transportable [123,124]. This system has been extensively used to investigate the behavioral responses of various mosquito species in Thailand and elsewhere in Asia [125-128]. Additionally, a more compact, modular assay system called the High Through-put Screening System (HITSS) was developed for mass screening of chemicals and adult mosquito responses; including contact irritancy, spatial repellency, and toxicity depending on the specific design set-up [129]. This modular system is of a reduced size compared to previous excito-repellency box devices and greatly minimizes the treated surface area required thereby reducing the amount of chemical required for handling and testing. This system is now approved by WHOPES for testing efficacy of new active ingredients intended for spatial repellent [45].

To better approximate insect behaviors in natural field settings, numerous experiments have been made over many decades using specially constructed experimental huts [4,31,121,130-136]. Most experimental hut studies have been conducted to observe the behavior of *Anopheles* mosquitoes; however, Grieco *et al*. [4] successfully demonstrated all 3 chemical actions could be observed in experimental huts using *Ae. aegypti* as a model system. The results obtained from both laboratory and field studies can help facilitate the choice of the most effective chemicals and measures to control house-frequenting adult mosquitoes. The behavioral responses of *Anopheles* species to various compounds are provided in Table 6 and those for *Aedes aegypti* and *Culex quinquefasciatus* in Table 7.

**Table 6:** **Evidence of behavioral avoidance responses to insecticides in*Anopheles*populations in Thailand (2000–2011)**

**Species**	**Field/Lab**	**Dose**	**Insecticide**	**Location**	**Published source**
				**(province-district)**	
*Anopheles minimus* A*	Lab	2.00 g/m^2^	DDT	Phrae-Rong Klang	Chareonviriyaphap *et al.* 2001 [125]
		0.0625 g/m^2^	Deltamethrin	Phrae-Rong Klang	
		0.0369 g/m^2^	Lambdacyhalothrin	Phrae-Rong Klang	
*Anopheles minimus* complex	Field	2.00 g/m^2^	DDT	Kanchanaburi-Pu Teuy	
		0.0625 g/m^2^	Deltamethrin	Kanchanaburi-Pu Teuy	
		0.0369 g/m^2^	Lambdacyhalothrin	Kanchanaburi-Pu Teuy	
*Anopheles minimus* A*	Lab	0.02 g/m^2^	Deltamethrin	Phrae-Rong Klang	Chareonviriyaphap *et al.* 2004 [70]
*Anopheles dirus* B†	Lab	0.02 g/m^2^	Deltamethrin	Chantaburi-Ban Paung	
*Anopheles minimus* complex	Field	0.02 g/m^2^	Deltamethrin	Kanchanaburi-Pu Teuy	
*Anopheles maculatus* B‡	Lab	0.02 g/m^2^	Deltamethrin	Tak-Mae Sot	
*Anopheles swadwongporni*	Field	0.02 g/m^2^	Deltamethrin	Kanchanaburi-Pu Teuy	
*Anopheles dirus* complex	Field	0.02 g/m^2^	Deltamethrin	Kanchanaburi-Pu Teuy	
*Anopheles minimus* A*	Field	2.00 g/m^2^	DDT	Tak-Mae-Sot	Potikasikorn *et al*. 2005 [127]
		0.02 g/m^2^	Deltamethrin	Tak-Mae-Sot	
		0.03 g/m^2^	Lambdacyhalothrin	Tak-Mae-Sot	
*Anopheles minimus* C§	Field	2.00 g/m^2^	DDT	Kanchanaburi-Pu Teuy	
		0.02 g/m^2^	Deltamethrin	Kanchanaburi-Pu Teuy	
		0.03 g/m^2^	Lambdacyhalothrin	Kanchanaburi-Pu Teuy	
*Anopheles maculatus*	Field	2.00 g/m^2^	DDT	Kanchanaburi-Pu Teuy	Muenworn *et al*. 2006 [137]
		0.5 g/m^2^	Permethrin	Kanchanaburi-Pu Teuy	
*Anopheles swadwongporni*	Field	2.00 g/m^2^	DDT	Kanchanaburi-Pu Teuy	
		0.5 g/m^2^	Permethrin	Kanchanaburi-Pu Teuy	
*Anopheles minimus* A*	Field	0.04 g/m^2^	Cypermethrin	Kanchanaburi-Pu Teuy	Pothikasikorn *et al*. 2007 [138]
		0.04 g/m^2^	Carbaryl	Kanchanaburi-Pu Teuy	
		0.19 g/m^2^	Malathion	Kanchanaburi-Pu Teuy	
*Anopheles minimus* C§	Field	0.04 g/m^2^	Cypermethrin	Kanchanaburi-Pu Teuy	
		0.04 g/m^2^	Carbaryl	Kanchanaburi-Pu Teuy	
		0.19 g/m^2^	Malathion	Kanchanaburi-Pu Teuy	
*Anopheles harrisoni*	Field	0.03 g/m^2^	Bifenthrin	Kanchanaburi-Pu Teuy	Tisgratog *et al*. 2011 [139]
*Anopheles minimus*	Field	0.03 g/m^2^	Bifenthrin	Tak-Mae Sot	

With the exception of one population (*An. minimus* s.s. in Tak Province/Mae Sot District to DDT) all species show contact excitation (irritancy) as the predominant response versus noncontact spatial repellency.

*Published as *Anopheles minimus* A, now formally named *Anopheles minimus* s.s.; †*Anopheles dirus* B now as *Anopheles crascens*; ‡*Anopheles maculatus* B as *Anopheles maculatus* s.s. [66] and § *Anopheles minimus* C as *Anopheles harrisoni.*

**Table 7:** **Evidence of behavioral avoidance responses in*Aedes aegypti*and*Culex quinquefasciatus*to insecticides in Thailand (2000 – 2011)**

**Species**	**Field/Lab**	**Dose**	**Insecticide**	**Location**	**Published source**
*Aedes aegypti*	Field	0.02 g/m^2^	Deltamethrin	Bangkok	Kongmee *et al*. 2004 [126]
*Aedes aegypti*	Field	0.02 g/m^2^	Deltamethrin	Pathum Thani	
*Aedes aegypti*	Field	0.02 g/m^2^	Deltamethrin	Nonthaburi	
*Aedes aegypti*	Lab	0.02 g/m^2^	Deltamethrin	Ayutthaya	
*Aedes aegypti*	Lab	0.02 g/m^2^	Deltamethrin	Bangkok	
*Aedes aegypti*	Lab	0.25 g/m^2^	Permethrin	Nonthaburi	Paeporn *et al.* 2007 [97]
*Aedes aegypti*	Field	0.025 g/m^2^	Alphacypermethrin	Kanchanaburi	Thanispong *et al.* 2009 [140]
		2.00 g/m^2^	DDT	Kanchanaburi	
*Aedes aegypti*	Field	0.025 g/m^2^	Alphacypermethrin	Chiang Mai	
		2.00 g/m^2^	DDT	Chiang Mai	
*Aedes aegypti*	Lab	0.025 g/m^2^	Alphacypermethrin	USDA	
		2.00 g/m^2^	DDT	USDA	
*Aedes aegypti*	Field	0.010%	Deltamethirn	Kanchanaburi	Mongkalagoon et al. 2009 [141]
		0.0113%	Cyphenothrin	Kanchanaburi	
		2.091%	d-Tetramethrin	Kanchanaburi	
		2.377%	Tetramethrin	Kanchanaburi	
*Aedes aegypti*	Field	0.05%	Alphacypermethrin	Chiang Mai	Thanispong *et al*. 2010 [142]
		0.05%	Deltamethrin	Chiang Mai	
		0.25%	Permethrin	Chiang Mai	
		4.00%	DDT	Chiang Mai	
*Aedes aegypti*	Field	0.05%	Alphacypermethrin	Kanchanaburi	
		0.05%	Deltamethrin	Kanchanaburi	
		0.25%	Permethrin	Kanchanaburi	
		0.25%	DDT	Kanchanaburi	
*Aedes aegypti*	Field	0.05%	Alphacypermethrin	Khon Kean	
		0.05%	Deltamethrin	Khon Kean	
		0.25%	Permethrin	Khon Kean	
		4.00%	DDT	Khon Kean	
*Aedes aegypti*	Field	0.05%	Alphacypermethrin	Nonthaburi	
		0.05%	Deltamethrin	Nonthaburi	
		0.25%	Permethrin	Nonthaburi	
		4.00%	DDT	Nonthaburi	
*Aedes aegypti*	Field	0.05%	Alphacypermethrin	Songkhla	
		0.05%	Deltamethrin	Songkhla	
		0.25%	Permethrin	Songkhla	
		4.00%	DDT	Songkhla	
*Aedes aegypti*	Field	0.05%	Alphacypermethrin	Satun	
		0.05%	Deltamethrin	Satun	
		0.25%	Permethrin	Satun	
		4.00%	DDT	Satun	
*Culex quinquefasciatus*	Field	0.02 g/m^2^	Deltamethrin	Nonthaburi	
		0.20 g/m^2^	Fenitrothion	Nonthaburi	
		0.20 g/m^2^	Propoxur	Nonthaburi	Sathantriphop *et al.* 2006 [113]
*Culex quinquefasciatus*	Field	0.02 g/m^2^	Deltamethrin	Tak	
		0.20 g/m^2^	Fenitrothion	Tak	
		0.20 g/m^2^	Propoxur	Tak	
*Culex quinquefasciatus*	Lab	0.02 g/m^2^	Deltamethrin	Nonthaburi	
		0.20 g/m^2^	Fenitrothion	Nonthaburi	
		0.20 g/m^2^	Propoxur	Nonthaburi	

All species show contact excitation (irritancy) as the predominant response versus noncontact spatial repellency.

### Behavioral avoidance to insecticides

#### DDT

Unlike physiological resistance, accurately measuring behavioral responses remains elusive and difficult to detect. Some agricultural and medically important insects, including malaria vectors, have allegedly demonstrated what has been termed “behavioral resistance” following repeated exposure to sub-lethal concentrations of DDT [30]. However, “behavioral avoidance” is preferred terminology as it is an innate, involuntary response to an external stimuli rather than a permanent, genetically-based shift in behavior as the development of apparent fixed behavioral changes because of insecticide selective pressure has not been sufficiently documented to occur under natural conditions [143]. The early observations on behavioral responses of mosquitoes focused on DDT and have been investigated using experimental huts and excito-repellency box (ERB) test systems. The first study on the irritant effect of DDT residual deposits was conducted using *Anopheles quadrimaculatus* where females were found to be irritated shortly after making contact with the treated surfaces resulting in a rapid escape response from a treated house before taking a blood meal [144]. Subsequent observations found that *An. quadrimaculatus* often received a lethal dose from this brief contact and perished within 24 hours [145]. Unfortunately, these studies were conducted without having a matched control (untreated houses) for comparison. Furthermore, the high mortality seen with *An. quadrimaculatus* may have been caused by further contact with toxic ingredients while attempting to leave a treated house through the small portals of the experimental hut [143]. In studies with *Anopheles albimanus* in Panama, Trapido [146] concluded that wild-caught mosquitoes lacking re-exposure to DDT for a long period of time, showed the same susceptibility levels to DDT as those from a laboratory colony with no history of previous exposure. Malaria vectors in some countries (e.g., Brazil, Thailand) have apparently never developed resistance to DDT [125,130] despite years of extensive use inside houses, suggesting that the particular mosquito populations avoid making direct physical contact with the chemical, thereby precluding any selection for resistance. Table 6 lists *Anopheles* species and levels of behavioral responses to DDT and synthetic pyrethroids in Thailand.

In Thailand, the irritant and repellent effects of DDT were demonstrated against two populations of *Anopheles minimus* (sensu lato) using an ERB test system [125]. A study comparing 2 members of the Minimus Complex; *An. minimus* (species A) and *Anopheles harrisoni* (species C) found that DDT produced a rapid and striking irritant response in both species, while repellency was more pronounced in *An. minimus*[127]. With *Ae. aegypti*, it has been suggested an association exists between increased levels of physiological resistance to DDT and a greater suppression of both excitation and repellency responses, yet the overall avoidance behavior remains a significant response compared to non-exposed controls [140].

### Pyrethroids

Numerous studies have found that synthetic pyrethroids clearly elicit a range of excitation and repellent effects on many insect species that also typically result in mosquitoes moving away ('avoidance’) from insecticide sprayed surfaces [30,147,148]. The extensive and continuing use of pyrethroids should serve as an incentive to intensify investigations on the operational significance of pyrethroid-induced avoidance behavior in mosquito vectors and other arthropods. Given the important role of indoor residual spraying of homes as a means of reducing risk of malaria transmission, the consequence of excitation and repellent actions should be well defined for specific malaria vectors in defined locations before and after beginning any large scale control program. Following the refinement of the ERB test system and development of the HITSS assay, both that allow the discrimination of 2 primary types of behavioral actions [12], a series of important findings on excito-repellency behavior in *Anopheles* mosquitoes have been reported in Thailand [19,70,125,127,137,138,142]. In general, synthetic pyrethroids consistently result in significantly stronger contact irritant responses in *Anopheles* compared to repellency (Table 6). For example, lambda-cyhalothrin and deltamethrin act as strong excitatory agents on test populations of *An. minimus* while showing relatively weak repellent activity [125]. Pothikasikorn *et al*. [127] also confirmed that *An. minimus* and *An. harrisoni* showed a rapid escape response to contact with lambda-cyhalothrin and deltamethrin, while repellency remained significant but lagged in intensity. Chareonviriyaphap *et al*. [70] described the excito-repellent actions of deltamethrin in four different *Anopheles* species, all representing important malaria vectors in Thailand, showing deltamethrin can produce a pronounced irritant response relative to a slower, weaker spatial repellency. Although repellency was less outstanding an escape response compared to irritancy, the repellent action and escape responses were still statistically significant compared to the matched paired controls (minus active ingredient).

Numerous behavioral responses of *Ae. aegypti* populations exposed to a series of pyrethroids (deltamethrin, permethrin, alpha-cypermethrin, cyphenothrin, d-tetramethrin and tetramethrin) have been investigated in depth in Thailand (Table 7). In general, all tested populations have exhibited moderate to strong irritancy when compared to repellency [97,101,129,140,142]. Far fewer numbers of populations of *Cx. quinquefasciatus* have been tested against the 3 principal classes of insecticides used in vector control; pyrethroids (deltamethrin), organophosphates (fenitrothion) and carbamates (propoxur), yet again, contact excitation appears stronger than repellency against all three compounds (Table 7). However, marked differences in behavioral responses have been seen between different mosquito populations, active ingredients and concentrations. Prominent irritant responses were observed in a long-established colony population exposed to deltamethrin and fenitrothion as compared to two field populations of the same species [106], demonstrating the importance of testing field populations as opposed to long-colonized material.

The behavioral responses to insecticides by mosquitoes are important components of a chemical’s overall effectiveness and value in reducing human-vector contact and disease transmission. To date, there is no convincing evidence of fixed behavioral 'resistance’ developing in mosquito species to continuous or intermittent exposure to insecticides [149]. Rather, the majority of data supports that a mosquito’s actions are part of an innate involuntary behavioral repertoire involving reflex stimulus–response mechanisms. Behavioral responses can be split into 2 distinct categories, stimulus-dependent and stimulus-independent actions [150]. The term *avoidance behavior* is generally used to describe actions that are stimulus-dependent by some combination of excitation and repellency [131].

A stimulus-dependent action requires sensory stimulation of the insect in order for an avoidance behavior to occur. In general, this form of avoidance enables the insect to detect a chemical after making direct contact but before acquiring a lethal dose [143] or detection of vapor-phase molecules of the active ingredient in the air that initiates repellency (deterrence). On the other hand, a stimulus-independent response does not require direct sensory stimulation of the insect for avoidance to occur but rather involves other natural behavioral components such as exophily (outside resting) or zoophily (non-human blood preference) in which an insect avoids the exposure to a chemical by preferentially utilizing habitats without insecticides present [151]. This second type of response has also been included as innate, genetically-driven phenotypic and genotypic behaviors [152]. Stimulus-dependent behavioral responses include the avoidance behaviors discussed in this review.

## Conclusions

A number of synthetic pyrethroids, i.e. allethrin, deltamethrin, permethrin, cypermethrin, alpha-cypermethrin, and cyfluthrin, are commonly used by home owners, private business and in the public sector to control both household pests and medically important insect vectors. In the past, the prevailing practice has been to classify chemicals simply based on their toxicity (killing) profile of insects. We have preferred the use of the term “chemical” in place of “insecticide” as it is more appropriate for recognizing the multiple actions these active ingredients have on mosquitoes via toxicity alone. This also recognizes that chemicals protect humans from the mosquito blood feeding by at least 3 different primary actions: excitation ('irritancy’), repellency and toxicity [4,32]. Historically, the vast majority of studies have focused on the direct toxicological (mortality) responses (susceptibility and resistance patterns) of chemicals on mosquito populations; whereas very little emphasis was placed on the vector's behavior in response to sub-lethal exposures to various compounds. Knowledge of a mosquito’s normal and chemical-induced behavioral responses under varying conditions (e.g., age, physiological state, parity, etc.) is important in the prioritization and design of appropriate vector prevention and control strategies. The development of insecticide resistance in insect pests and disease vectors worldwide is increasing on an alarming scale. However, patterns of resistance is not uniform across all areas, with some vector populations having had low instances of resistance in spite of long-term use of chemicals used to control them, suggesting that behavioral responses may likely be playing a significant role influencing how certain chemicals perform to interrupt human-vector contact while reducing the selection pressure on target insects for developing physiological resistance [32]. In Thailand, populations of *Ae. aegypti* showing patterns of low-grade resistance to pyrethroids may indicate that resistance is increasing in a particular population; it is also possible that resistance may be declining subsequent to diminution of selective pressure. We postulate that behavioral avoidance may result in a third alternative - a low level equilibrium of physiological resistance.

Avoidance responses in which normal host-seeking and biting activities are disrupted pose a challenge to wild adult mosquitoes on a tight energy budget to fly longer and greater distances than would have otherwise occurred. This might have an impact on its survival or fecundity thereby impacting both subsequent vector population densities and disease transmission. Behavioral avoidance of insecticides may have similar effects, should insecticide coverage be sufficiently high. Recent evidence of apparent decreasing densities of vectors and reduction of malaria in Africa poses questions as to the reasons or mechanisms behind the population reductions [153]. The debate whether repellency or toxicity is the more favored attribute of an insecticide [32,33,154], may likely depend on the specific vector species being targeted for control. As IRS and ITN chemical treatments gradually reach sublethal doses what are the implications on the biology of mosquito vectors, their populations and the diseases they transmit? Is behavioral avoidance a liability or an asset in the control of new infections? For example, ITNs have been implicated in the massive reduction in *An. darlingi* in Suriname [155], whether due to direct killing effects or deterrence. On the other hand, deterrence may impact transmission dynamics that possibly exacerbate control efforts to suppress transmission [156,157]. One example of attempting to expolit contact irritant responses of *Ae. aegypti* has been the evaulation of sublethal concentrations and focal application of pyrethroid chemicals for the distruption of normal blood feeding and resting behavior [25].

Whether a chemical is acting by direct contact with a mosquito or spatially from a distance, both behavioral responses are stimulus–response actions that result in distinct movement away from an area where the chemical is present. We now have various types of excito-repellency test systems (e.g., ERB and HITSS assays) that can accurately measure the behavioral responses of mosquitoes to chemicals. WHOPES has very recently recommended a battery of test procedures to better address the efficacy and mode of action of active ingredients intended for “spatial repellent” applications [158]. However, the “proof of principle” that spatial repellency can effectively reduce malaria or dengue transmission, both indoors and out, merits further investigation.

To conclude, we believe that a better understanding and evaluation of physiological and behavioral responses by mosquitoes to chemicals are an important, if not critical, components of any disease control operation dependent on this abatement strategy. We recommend that in addition to establishing a country-wide network of routine resistance monitoring and behavioral response evaluations where vector control is taking place, a greater emphasis is needed to investigate the biochemical and genetic mechanisms responsible for resistance in vector populations. There should also be an expansion of the number of different vector species examined. For example, there have been virtually no investigations on the response to insecticides by the primary culicine vectors of Japanese encephalitis virus or Mansonia species responsible for lymphatic filariasis. Lastly, because of the recent governmental re-organization in Thailand allowing each municipality decision-making authority for how local vector control operations are conducted, including the purchasing insecticides, there is concern that both ineffectual and inefficient vector control practices might result. Such an unstructured and independently managed system might also contribute to an escalation of insecticide resistance. It would be advisable, that clear and enforceable policies that are evidence-based for insecticide selection be directed from the MOPH, Department of Disease Control.

Knowledge of vector/pest susceptibility to pesticides, changing patterns of resistance and their operational implications, will continue to be basic requirements to help guide chemical use in evidence-based, vector-borne disease and pest control programs. Although not an exhaustive compilation, this review has attempted to summarize the current knowledge of how chemicals affect various vector species and populations in Thailand. With this background, it is hoped that operational and research endeavors will benefit from this overview and focus on areas where critical information is lacking.

## Abbreviations

IRS: Indoor residual spraying; ITN: Insecticide-treated netting (bednets); DEET: *N*, *N*-diethyl-*meta*-toluamide; PCO: Pest control operators; MFO: Mixed function oxidases (P-450 mediated monooxygenases); GST: Glutathione *S*-transferases; ERB: Excito-repellency box; HITSS: High throughput screening system; WHOPES: World Health Organization Pesticide Evaluation Scheme; MOPH: Ministry of Public Health (Thailand).

## Competing interests

The authors declare that they have no competing interests.

## Authors’ contributions

TC reviewed the literature and formulated the review. TC wrote the paper with assistance from MJB, WS, MK, VC, and RNK. All authors read and approved the final manuscript.
